# Recovery of Biologically Active Compounds from Stinging Nettle Leaves Part II: Processing of Exhausted Plant Material after Supercritical Fluid Extraction

**DOI:** 10.3390/foods12040809

**Published:** 2023-02-14

**Authors:** Saša Đurović, Lato Pezo, Uroš Gašić, Stanislava Gorjanović, Ferenc Pastor, Julia G. Bazarnova, Yulia A. Smyatskaya, Zoran Zeković

**Affiliations:** 1 Laboratory of Chromatography, Institute of General and Physical Chemistry, Studentski trg 12/V, 11158 Belgrade, Serbia; 2 Graduate School of Biotechnology and Food Industries, Peter the Great Saint-Petersburg Polytechnic University, Polytechnicheskaya Street, 29, 195251 Saint-Petersburg, Russia; 3 Institute for Biological Research “Siniša Stanković”—National Institute of Republic of Serbia, University of Belgrade, Bulevar despota Stefana 142, 11060 Belgrade, Serbia; 4 Faculty of Chemistry, University of Belgrade, Studentski trg 12, 11000 Belgrade, Serbia; 5 Faculty of Technology, University of Novi Sad, Bulevar Cara Lazara 1, 21000 Novi Sad, Serbia

**Keywords:** stinging nettle leaves, exhausted plant material, HPLC-DAD-MS/MS, polyphenolic profile, biological activity, ANN modeling

## Abstract

Stinging nettle (*Urtica dioica* L.) is one fantastic plant widely used in folk medicine, pharmacy, cosmetics, and food. This plant’s popularity may be explained by its chemical composition, containing a wide range of compounds significant for human health and diet. This study aimed to investigate extracts of exhausted stinging nettle leaves after supercritical fluid extraction obtained using ultrasound and microwave techniques. Extracts were analyzed to obtain insight into the chemical composition and biological activity. These extracts were shown to be more potent than those of previously untreated leaves. The principal component analysis was applied as a pattern recognition tool to visualize the antioxidant capacity and cytotoxic activity of extract obtained from exhausted stinging nettle leaves. An artificial neural network model is presented for the prediction of the antioxidant activity of samples according to polyphenolic profile data, showing a suitable anticipation property (the *r*^2^ value during the training cycle for output variables was 0.999).

## 1. Introduction

*Urtica dioica* L. (commonly known as stinging nettle) is an extraordinary plant from Urticaceae botanical family [1]. This unique plant has been widely used in folk medicine, the cosmetic industry, pharmacy, and food [2]. Stinging nettle leaves have a long history as a remedy for flailing arthritis or paralytic limbs, the stimulation of circulation, and the warming of the joints and extremities (urtication) [3]. In addition, it is used to treat different diseases and disorders, such as gout, eczema, and urinary, bladder, and kidney problems [4]. Several studies have reported a wide range of biological activities, e.g., antioxidant, antimicrobial, anti-inflammatory, anti-ulcer, and analgesic properties [3,5,6]. All these activities can be ascribed to the chemical composition of this plant. Various studies reported the presence of polyphenolic compounds; vitamins B, K, and C; terpenoids; fatty acids; minerals (especially iron); carotenoids; chlorophyll; essential amino acids; tannins; carbohydrates; sterols; polysaccharides; and isolectins [2,6,7,8,9,10,11,12,13]. Among these compounds are compounds necessary for the human diet. Thus, this plant is also used for the preparation of many dishes, such as omelets, soups, rice, salads, noodles [14], and even bread [15].

Several extraction techniques have been developed to isolate desired compounds from the original matrix. Generally, they can be divided into two major groups: conventional (hydrodistillation, maceration, Soxhlet extraction, etc.) and nonconventional (ultrasound-assisted, microwave-assisted, supercritical fluid, and subcritical water extraction) techniques [6]. Conventional extraction techniques usually require large portions of organic solvents, which are toxic and flammable, time consumption, and labor [16]. Nonconventional techniques have been developed in order to overcome all drawbacks of conventional techniques. Ultrasound-assisted (UAE) and microwave-assisted (MAE) techniques use ultrasonic power and microwaves, respectively, to initiate and facilitate the diffusion of biologically active molecules from their natural sources [6]. These techniques are widely used to extract various organic compounds from their sources.

This study is a continuation of the previously published research by Đurović et al. [17]. In that study, the authors investigated extracts obtained after supercritical fluid extraction (SFE) of stinging nettle leaves under different extraction conditions. Exhausted plant material was collected and subsequently extracted with UAE and MAE, which is the main subject of this article. The obtained UAE and MAE extracts of the exhausted leaves were analyzed to determine the total extraction yield and polyphenolic profile. Furthermore, the extracts were assessed for biological activity, i.e., antioxidant, cytotoxic, and antimicrobial activities. Results were compared with those obtained for nontreated stinging nettle leaves extracts using UAE and MAE. A pattern recognition technique (such as principal component analysis (PCA)) was used for the antioxidant capacity and cytotoxic activity dataset (which were used as descriptors) to characterize and differentiate among extracts obtained from exhausted stinging nettle leaves. This research also investigated the possibility of predicting the antioxidant activity of samples using an artificial neural network model.

## 2. Material and Methods

### 2.1. Plant Material

In 2015, during the months of April and May, the leaves of stinging nettle (*Urtica dioica* L.) were gathered in the Vršac region of Southeastern Banat in the Autonomous Province of Vojvodina, Republic of Serbia. The leaves were dried in the shade for a month without artificial means. The dried leaves were then blended and stored in paper bags until use. A reference specimen (*Urtica dioica* L., from the Vršac area, collected by Saša Đurović, N° 2-1539) has been kept at the Herbarium BUNS, University of Novi Sad, Faculty of Science, Department of Biology and Ecology.

### 2.2. Extraction Procedures

Exhausted plant material after SFE extraction [17] (conditions are given in Table 1) was used for the UAE and MAE extractions.

The UAE extraction was conducted under the ultrasonic power of 156 W, while the MAE was conducted using the microwave power of 450 W according to previously described procedures [6]. In both cases, extractions were conducted for 30 min using 5 g of plant material and 150 mL of water (solvent-to-sample ratio 30:1). After completing the extraction, the extracts were promptly filtered through a Whatman No. 1 filter paper under vacuum. They were then transferred to the glass bottles and stored at 4 °C to prevent degradation. The extraction yield was determined by taking 10 mL of the liquid extract, evaporating the solvent under vacuum, drying at 110 °C for 3 h, cooling for 20 min, and measuring. The total extraction yield was expressed in grams per 100 g of SN leaves (g/100 g SN).

### 2.3. Total Phenolics and Flavonoids Contents

Total phenolic (TPC) and total flavonoid (TFC) contents were determined using the previously described spectrophotometric methods [18,19]. Briefly, for the determination of TPC, the extract was mixed with 7.9 mL of water and Folin–Ciocalteu’s reagent and 1.5 mL of 20% Na_2_CO_3_. The absorbance was measured at 750 nm (VIS spectrophotometer; Janwey 6300, Germany) after 1 h against the blank solution (prepared in a similar manner using distilled water instead of the extract).

For the determination of TFC, the extract was mixed with 1 mL of distilled water and 2.5 mL of AlCl_3_ solution. A blank solution was prepared using distilled water instead of the analyzed extract. The absorbances of the tested solutions against the blank were measured immediately at 430 nm. Results were expressed as mg CAE/g SN (milligrams of chlorogenic acid equivalents per gram of stinging nettle leaves) and mg CE/g SN (milligrams of catechin equivalents per gram of stinging nettle leaves) for TPC and TFC, respectively.

### 2.4. Polyphenolic Profile

The polyphenolic profile of the obtained extract was investigated according to the previously described procedure [20] using a Dionex Ultimate 3000 UHPLC system equipped with a diode array detector (DAD) connected to a TSQ Quantum Access Max triple-quadrupole mass spectrometer (Thermo Fisher Scientific, Basel, Switzerland). The elution was performed at 40 °C using a Syncronis C18 column (100 × 2.1 mm, 1.7 m particle size). The mobile was a mixture of water plus 0.01% acetic acid (A) and acetonitrile (B), applying the following gradient elution: 5% B in the first 2.0 min, 5–95% B from 2.0 to 12.0 min, 95% to 5% B from 12.0–13.0 min, and 5% B until the 20th min. The flow rate was 0.3 mL/min with an injection volume of 5 µL. The detection wavelengths were 254 and 280 nm.

Qualitative and quantitative analyses were conducted using an MS/MS detector. The mass spectrometry operated in the negative ionization mode. The operational *m*/*z* range was 100–1000. The two MS^2^ fragments for each compound were used for the time-selected reaction monitoring (tSRM) experiments. The used fragments were previously marked as dominant in the product ion scanning (PIS) experiments. The final results were expressed as milligrams of analyzed compound per liter of extracts (mg/L).

### 2.5. Biological Activity

Antioxidant activity was assessed by three previously described in vitro spectrophotometric methods: DPPH radical scavenging activity [21], reducing power assay [22], and direct current (DC) polarographic assay (HMPC) based on the decrease of anodic current of HydroxoPerhydroxoMercury(II) complex [23]. The results for the DPPH assay were expressed as IC_50_ values (µg/mL), for reducing power assay as EC_50_ (µg/mL), while the results of the HPMC method were expressed in %/mL.

The antibacterial activity was tested against the *Staphylococcus aureus* ATCC 25923, *Escherichia coli* ATCC 25922, *Klebsiella pneumonia* ATCC 13883, *Proteus vulgaris* ATCC 13315, *Proteus mirabilis* ATCC 14153, and *Bacillus subtilis* ATCC 6633, while the antifungal activity was tested against the *Aspergillus niger* ATCC 16,404 and *Candida albicans* ATCC 10231 according to the previously described procedure [23]. The results were expressed as a minimal inhibitory concentration (MIC) in µg/mL.

Cytotoxic activity was determined against the three different cell lines: RD (cell line derived from human rhabdomyosarcoma), Hep2c (cell line derived from human cervix carcinoma–HeLa derivative), and L2OB (cell line derived from murine fibroblast), using the previously described MTT (3-[4,5-dimethylthiazol-2-yl]-2,5-diphenyl tetrazolium bromide) assay [24]. The results were expressed as IC_50_ (µg/mL), which represents the concentration of an agent inhibiting cell survival by 50% compared with vehicle-treated control.

### 2.6. ANN Modeling

For the prediction of the antioxidant activity of the tested extracts, artificial neural network (ANN) modeling was used in combination with a multi-layer perceptron model (MLP) with three layers. The experimental data were normalized, and the Broyden–Fletcher–Goldfarb–Shanno (BFGS) algorithm was used during the network modeling [25]. During the ANN modeling, different topologies were studied by changing the number of neurons in the hidden layer and randomized initial weights and biases [26].

Yoon’s interpretation method showed the relative influence of phenolic component content on the antioxidant activity of the samples [27].

### 2.7. Statistical Analyses

All measurements were performed three times, and the results are given as the mean ± standard deviation (SD). To compare the differences between the samples, a post-hoc Tukey’s HSD test was used at a significance level of *p* < 0.05. The data were analyzed using PCA (principal component analysis) to examine the relationships between the variables and group the objects. The analysis was conducted using the STATISTICA 10.0 software (StatSoft Inc., Tulsa, OK, USA).

## 3. Results and Discussion

### 3.1. Extraction Yield

After finishing the extraction procedures, the next step was evaporating the solvent and determining the extraction yield. Results for both techniques are given in Table 2. The obtained results indicated different behavior of exhausted plant material compared to the starting plant material before SFE. Such differences are clearly in close connection with the conditions of the supercritical fluid extraction, i.e., pressure and temperature at which plant material was exposed before UAE and MAE. The highest yield was achieved in sample 5 for UAE and sample 3 in the case of the MAE technique.

In the case of the UAE technique, only the UAE-1 sample had a lower yield than starting material (UAE sample). At the same time, MAE-3 and MAE-4 achieved lower extraction yield than the starting material (MAE sample). For UAE, higher yields were achieved in the exhausted material exposed to higher pressure, while the MAE situation was quite the opposite, in which higher yields were achieved in the material exposed to lower pressure. The temperature had a different influence. Higher temperature positively influenced the yield in UAE at 100 bar (UAE-1 and UAE-2), then did the opposite, i.e., the lower temperature was better. MAE showed more dispersion of the results. The higher temperature gave better results when the material was exposed to the lower pressure (MAE-1 and MAE-2). It was also the same after the exposure to 300 bar, but after the extraction at 200 bar, the material exposed to the lower temperature showed better results.

It has already been shown that the MAE technique is more efficient than UAE [6,28,29]. In addition, it could also be noticed that, in all cases, MAE gave a higher yield than UAE. Therefore, these results are in accordance with the previous findings.

### 3.2. Polyphenolic Profile and Composition of the Extracts

After determining the extraction yield, the following stage of this study was to investigate the chemical profile, i.e., to determine the total content of polyphenolic compounds and flavonoids. These analyses were performed using spectrophotometric methods and the HPLC technique. The results of spectrophotometric methods are given in Table 3.

Results showed that, in both cases, MAE was a better extraction technique for isolating phenolic compounds and flavonoids. The results also showed completely different trends for UAE and MAE, as was the case for the extraction yield. The highest yield of phenolic compounds in UAE extracts was reached at 300 bar. However, the lower pressure was shown to be better in the case of MAE. The same trend was noticed for flavonoids.

Opposite to the spectrophotometric method, the HPLC analysis (chromatograms are shown in Figure S1, Supplementary Data) showed a completely different picture (Table 4). The contents of quantified phenolic compounds in both UAE and MAE extracts were significantly lower than that obtained from the starting material. The results also confirmed that MAE was a better technique for extracting phenolic compounds. The total yield was the highest in extracts obtained from the plant material after exposure to the lowest pressure during the supercritical fluid extraction (SFE) for both extraction techniques. The explanation may be that SFE is less selective when pressure increases and more compounds were extracted at 200 and 300 bar. In addition, differences between the results of the spectrophotometric methods and HPLC analysis were expected because the spectrophotometric test gave a positive response in the presence of many different classes of organic compounds, e.g., carbohydrates, amino acids, nucleotides, thiols, unsaturated fatty acids, proteins, vitamins, amines, aldehydes, and ketones [30,31,32].

HPLC analysis showed that, in both UAE and MAE extracts, phenolic acids are principal compounds. The main compound in UAE extract is *p*-coumaric acid, followed by caffeic and ferulic acids. The principal compound in MAE extract is caffeic acid. Besides caffeic acid, 5-*O*-caffeoylquinic acid, *p*-coumaric acid, and ferulic acid are present in higher amounts. The results showed that *p*-coumaric, *p*-hydroxyphenylacetic acid, and ferulic acid were better extracted from the exhausted plant material than the nontreated material in UAE. On the other hand, 5-*O*-caffeoylquinic, caffeic, and vanillic acids were better extracted from the nontreated plant material. In the case of MAE, the contents of caffeic acid, ferulic acid, and 5-*O*-caffeoylquinic acid were higher in extracts of nontreated plant material. In contrast, vanillic and *p*-hydroxyphenylacetic acids were higher in extracts obtained from exhausted plant material.

### 3.3. Biological Activity of Obtained Extracts

After the analysis of the chemical profile, all extracts were assessed for biological activity, i.e., antioxidant and cytotoxic activity. Antioxidant activity was analyzed using two different spectrophotometric and polarographic methods (Table 5). Cytotoxic activity was determined against three cell lines (Table 6).

The results for antioxidant activity showed different trends. Activity determined by the HPMC method increased and reached its maximal value in samples UAE-2 and UAE-3 and then decreased. The EC_50_ value decreased when compared with the value of extract obtained from nontreated leaves (UAE) and then reached a plateau. In the DPPH assay, the UAE-3 sample showed the highest activity, although the results were similar. In the case of MAE extracts, sample MAE-1 showed the highest potency in the HMPC method. Activity sharply decreased in sample MAE-2, then increased again (samples MAE-3 and MAE-4), and decreased again in samples MAE-5 and MAE-6. The DPPH assay and reducing power test potency of analyzed samples were similar. When comparing UAE and MAE techniques, MAE extracts showed higher potency, according to the previously obtained results [6].

The results for cytotoxic activity are presented in Table 6. The extracts obtained using both techniques were tested against three cell lines. From the present results, activity likely increased in both cases (UAE and MAE), and the highest potency was observed in samples UAE-6 and MAE-6. The most potent activity was observed against the RD cell line for both UAE-6 and MAE-6 extracts, while lesser activity was observed against the L2OB cell line. Taking into account that, according to the American National Cancer Institute (NCI), the criterion for cytotoxic activity of plant extract is IC_50_ < 30 µg/mL [33], the results indicated that most of the samples could be considered cytotoxic agents. It could also be noticed that all samples obtained after the extraction of exhausted material showed higher potency than extracts obtained using nontreated stinging nettle leaves.

The results of the correlation of the TPC and TFC biological activity are given in Table S1 (Supplementary Data). A negative correlation was observed between the HPMC and the other two antioxidant assays (IC_50_ and EC_50_, statistically significant at *p* < 0.01). The TPC and TFC assays were positively correlated and statistically significant at *p* < 0.001. These two assays were negatively correlated to IC_50_ (*p* < 0.001) and EC_50_ assays (*p* < 0.05), while the correlation to HPMC assays was positive (statistically significant at *p* < 0.01). The cytotoxic activity assays (Hep2c, RD, and L2OB) showed intensive mutual correlation (*p* < 0.001), but there was no statistically significant correlation to antioxidant assays nor a significant correlation to TPC and TFC assays.

Besides antioxidant and cytotoxic activities, the antimicrobial activity of all extracts was also assessed, and the results are summarized in Tables S2 and S3 (Supplementary Data). The results varied depending on the tested microbial strain and used extraction technique. In this particular case, UAE-6 and MAE-6 showed the highest potency, including cytotoxic activity. The results also showed that extracts from exhausted leaves were more potent than those from the nontreated plant material.

Obviously, the extracts obtained from the exhausted plant material had higher activity than those obtained from the nontreated plant material. Combining these results with those of the HPLC analysis implies that polyphenolic compounds are not the primary carrier of biological potency. The explanation may be the side reaction that occurred during the UAE and MAE, e.g., Maillard reaction and caramelization, at the elevated temperatures, resulting in the creation of new and more potent compounds, which also give a positive reaction with Folin reagent and increased total phenolic content [34,35].

### 3.4. PCA Analysis

**Figure 1:**
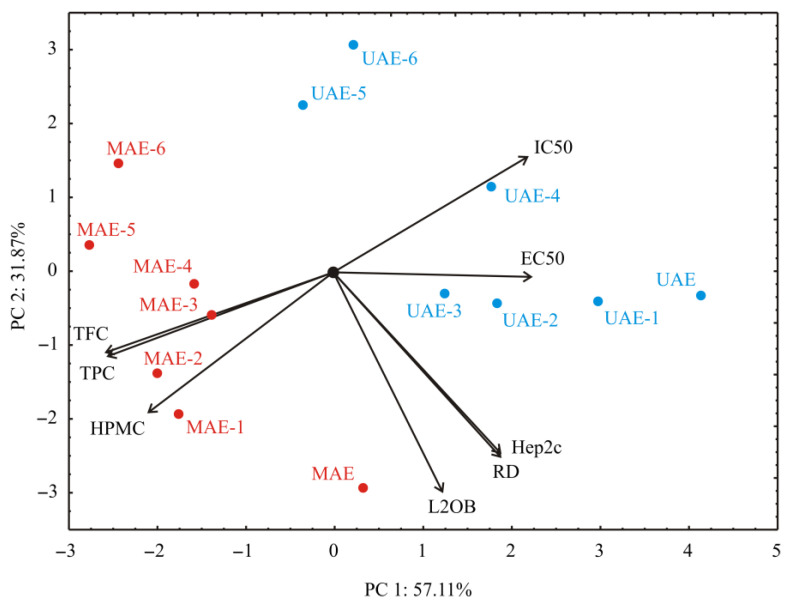
PCA ordination of variables based on component correlations.

The principal component analysis of biological activity assays for the tested extracts showed that two principal compounds explained 88.98% of the total variance in eight variables factor space (antioxidant and cytotoxic activity of extract), covering 58.11% and 31.87%, respectively. The calculated eigenvalues were 4.57 and 2.55, respectively. Based on the PCA analysis results, IC_50_, EC_50_, Hep2c, and RD displayed positive influence (Figure 1). They contributed 13.2%, 13.9%, 9.9%, and 9.9% of the total variance, respectively. The negative impact on the first principal component was noticed for assays: HPMC (12.2%), TFC (18.4%), and TPC (18.1%). The positive contribution for the second principal component was seen for the IC_50_ assay (9.0% of the total variance, based on correlations), while the influences of HPMC (11.9%) and the cytotoxic activity assays Hep2c (20.9%), RD (20.3%), and L2OB (29.9%) were negative.

The map of PCA revealed that the PC1 component explained the differentiation among the samples according to antioxidant assays: IC_50_, EC_50_, and HPMC, as well as TPC and TFC assays. At the same time, the PC2 coordinate showed variations in the cytotoxic activity of the samples (Hep2c, RD, and L2OB) between extracts obtained from exhausted leaves samples.

According to the PCA graphic, the most evident differentiation was observed between UAE and MAE samples. This difference was explained primarily based on the PC1 coordinate, which showed the difference in antioxidant capacity between the samples. The PC2 component showed variations in the cytotoxic activity of the samples. The MAE samples presented higher antioxidant capacity than the UAE samples, while longer-treated samples showed lower cytotoxic activity of the samples.

### 3.5. ANN Model

The BFGS 135 algorithm was successfully applied, while the exponential and identity functions were employed as hidden and output activation functions. The obtained ANN model was complex (306 weight-bias coefficients), explaining the high nonlinearity of the observed system. The ANN model predicted experimental values very close to the anticipated values in most cases (the obtained *r*^2^ values were 0.998, 0.999, and 0.999 for IC_50_, EC_50_, and HPMC assays, respectively). The optimal number of neurons in the ANN model for the prediction of antioxidant activity assays of extract obtained from exhausted leaves was 12 (network MLP 18-12-3), while the highest *r*^2^ values during the training cycle were 0.999.

**Figure 2:**
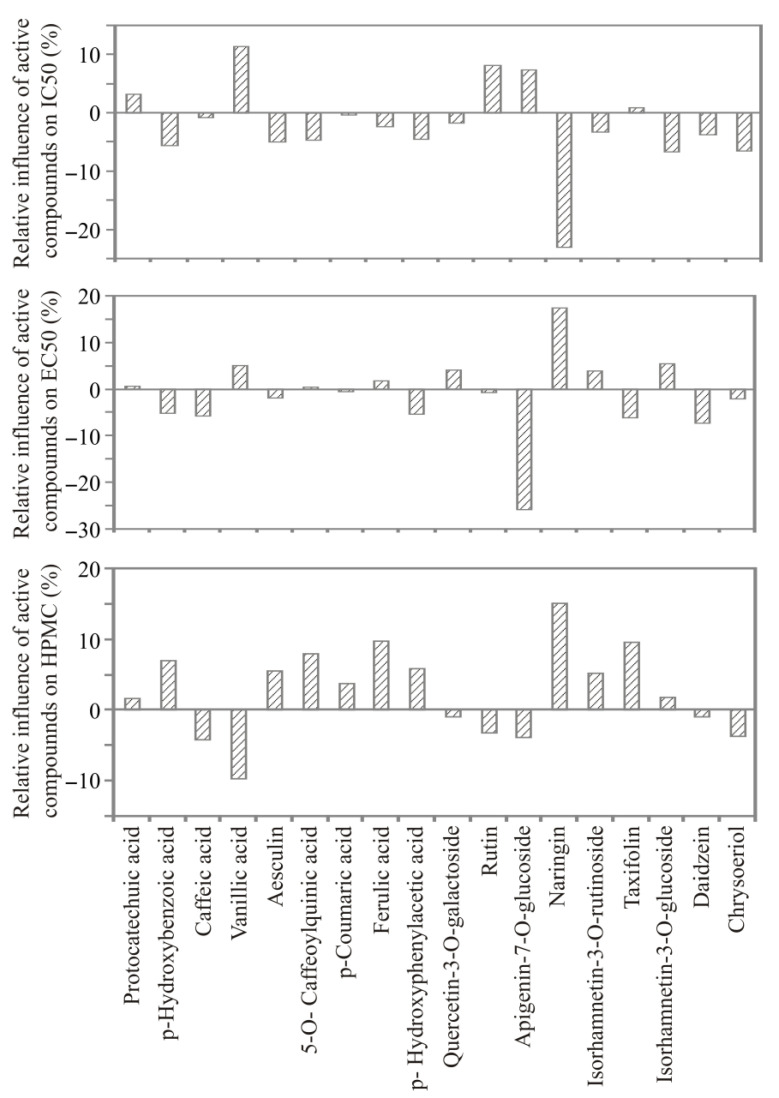
The relative importance of the polyphenols content on antioxidant activity assays of extract obtained from exhausted leaves.

According to Figure 2, vanillic acid, rutin, and apigenin-7-*O*-glucoside content were the most positively influential parameters for IC_50_ calculation, with an approximately relative importance of 7.35–11.48%, while the most negative influence variable was naringin, with the relative influence of −23.2%.

Apigenin-7-*O*-glucoside content was the most negatively influential parameter for EC_50_ calculation, with an approximately relative importance of −25.99%, while the most positive influence variable was naringin, with a relative influence of 17.43%.

Ferulic acid, naringin, and taxifolin content were the most positively influential parameters for IC_50_ calculation, with an approximately relative importance of 9.59–15.16%, while the most negative influence variable was vanillic acid, with a relative influence of −9.82%.

## 4. Conclusions

Exhausted plant material obtained after supercritical fluid extraction (SFE) was extracted using ultrasound-assisted and microwave-assisted extraction techniques. The results showed that a significant amount of biologically active compounds remained in the plant material after SFE. Although SFE extracted a wide range of compounds, the extracts obtained from the exhausted material were shown to be more potent in antioxidant, cytotoxic, and antimicrobial activity assays than the extracts prepared from the nontreated plant material. The results indicated that some of the polyphenolic compounds were extracted during the SFE, which is consistent with the results of the HPLC analysis. Based on those results, it might be concluded that new compounds with significant potency were created during the UAE and MAE extraction and that extraction of the compounds was more efficient after the SFE because plant cells would already be deformed at elevated pressure, causing easier diffusion. To summarize, a significant amount of the biologically active compounds remained in plant material after applying only one extraction technique. Therefore, several techniques should be applied for more efficient utilization of plant material.

## Figures and Tables

**Table 1:** Supercritical fluid extraction and separation conditions (data originated from [17]).

Parameter	Extracts					
	1	2	3	4	5	6
Pressure (bar)	100	100	200	200	300	300
Temperature (°C)	40	60	40	60	40	60
Density (g/cm^3^)	0.629	0.290	0.840	0.724	0.910	0.830
**Separation parameters**						
Pressure (bar)	15	15	15	15	15	15
Temperature (°C)	23	23	23	23	23	23

**Table 2:** Extraction yield of the obtained extract.

Sample	Yield (mg/mL)	Yield (g/100 g)
	UAE	
UAE	3.86 ± 0.06 ^b^	11.58 ± 0.09
UAE-1	3.06 ± 0.09 ^a^	9.18 ± 0.06
UAE-2	5.51 ± 0.07 ^c^	16.53 ± 0.10
UAE-3	6.09 ± 0.08 ^d^	18.27 ± 0.11
UAE-4	3.96 ± 0.04 ^b^	11.88 ± 0.08
UAE-5	8.40 ± 0.09 ^e^	25.20 ± 0.06
UAE-6	6.09 ± 0.03 ^d^	18.27 ± 0.11
	MAE	
MAE	10.27 ± 0.09 ^h^	30.81 ± 0.11
MAE-1	10.56 ± 0.10 ^i^	31.68 ± 0.12
MAE-2	10.79 ± 0.08 ^j^	32.37 ± 0.15
MAE-3	9.80 ± 0.06 ^g^	29.40 ± 0.10
MAE-4	9.65 ± 0.06 ^fg^	28.95 ± 0.09
MAE-5	10.74 ± 0.10 ^ij^	32.22 ± 0.09
MAE-6	9.58 ± 0.08 ^f^	28.74 ± 0.08

Values are means of three repeated experiments ± standard deviation. Values in the same column with the different superscript lowercase letters are statistically different (*p* < 0.05).

**Table 3:** Total phenolic and flavonoid contents.

Sample	TPC (mg CAE/g SN)	TFC (mg CE/g SN)
	**UAE**	
UAE	133.41 ± 1.26 ^b^	3.31 ± 0.11 ^b^
UAE-1	110.80 ± 2.22 ^a^	2.86 ± 0.09 ^a^
UAE-2	185.35 ± 1.52 ^d^	5.18 ± 0.16 ^d^
UAE-3	194.52 ± 2.82 ^e^	5.33 ± 0.09 ^d^
UAE-4	149.29 ± 2.23 ^c^	3.60 ± 0.08 ^c^
UAE-5	289.23 ± 3.11 ^g^	8.41 ± 0.06 ^f^
UAE-6	219.57 ± 2.65 ^f^	6.33 ± 0.11 ^e^
	MAE	
MAE	371.92 ± 3.56 ^j^	10.92 ± 0.10 ^h^
MAE-1	395.14 ± 2.99 ^k^	12.27 ± 0.06 ^j^
MAE-2	428.14 ± 3.59 ^m^	12.36 ± 0.08 ^j^
MAE-3	347.46 ± 4.11 ^i^	10.77 ± 0.09 ^h^
MAE-4	337.69 ± 3.25 ^h^	10.25 ± 0.08 ^g^
MAE-5	404.50 ± 3.77 ^l^	11.32 ± 0.07 ^i^
MAE-6	341.37 ± 2.85 ^hi^	10.96 ± 0.09 ^h^

Values are means of three repeated experiments ± standard deviation. Values in the same column with the different superscript lowercase letters are statistically different (*p* < 0.05).

**Table 4:** Phenolic profile of UAE and MAE extracts of exhausted plant material.

Compound	Content (mg/L)						
	UAE	UAE-1	UAE-2	UAE-3	UAE-4	UAE-5	UAE-6
Protocatechuic acid	4.50 ± 0.20 ^c^	2.32 ± 0.02 ^b^	2.26 ± 0.04 ^b^	ND *	ND	1.20 ± 0.06 ^a^	ND
*p*-Hydroxybenzoic acid	1.11 ± 0.06 ^a^	1.73 ± 0.01 ^d^	1.64 ± 0.06 ^c^	1.88 ± 0.09 ^e^	1.84 ± 0.02 ^e^	1.34 ± 0.05 ^b^	1.81 ± 0.03 ^e^
Caffeic acid	20.85 ± 0.52 ^g^	9.88 ± 0.03 ^e^	10.70 ±0.09 ^f^	4.04 ± 0.10 ^a^	6.67 ± 0.09 ^c^	4.84 ± 0.05 ^b^	6.91 ± 0.10 ^d^
Vanillic acid	1.05 ± 0.01 ^a^	1.96 ± 0.02 ^d^	1.74 ± 0.01 ^c^	1.95 ± 0.03 ^d^	2.05 ± 0.03 ^e^	1.33 ± 0.03 ^b^	2.01 ± 0.08 ^e^
Aesculin	1.96 ± 0.06 ^e^	0.69 ± 0.01 ^a^	0.88 ± 0.01 ^b^	0.96 ± 0.06 ^c^	1.15 ± 0.03 ^d^	0.69 ± 0.02 ^a^	1.10 ± 0.06 ^d^
5-*O*-Caffeoylquinic acid	31.00 ± 0.36 ^g^	0.54 ± 0.01 ^a^	1.58 ± 0.06 ^f^	0.63 ± 0.02 ^b^	1.40 ± 0.02 ^e^	0.77 ± 0.02 ^c^	1.21 ± 0.04 ^d^
*p*-Coumaric acid	4.60 ± 0.05 ^a^	9.72 ± 0.06 ^e^	8.70 ± 0.05 ^c^	12.04 ± 0.11 ^f^	8.80 ± 0.09 ^c^	7.55 ± 0.08 ^b^	9.17 ± 0.12 ^d^
Ferulic acid	1.71 ± 0.03 ^a^	3.58 ± 0.06 ^f^	2.88 ± 0.03 ^c^	3.16 ± 0.06 ^e^	2.84 ± 0.01 ^c^	2.11 ± 0.06 ^b^	2.97 ± 0.03 ^d^
*p*-Hydroxyphenylacetic acid	0.33 ± 0.00 ^b^	0.42 ± 0.01 ^c^	0.42 ± 0.01 ^c^	0.48 ± 0.02 ^d^	0.51 ± 0.01 ^e^	0.30 ± 0.01 ^a^	0.51 ± 0.02 ^e^
Quercetin-3-*O*-galactoside	0.05 ± 0.00 ^d^	ND	0.03 ± 0.00 ^c^	0.01 ± 0.00 ^a^	0.02 ± 0.00 ^b^	ND	0.03 ± 0.00 ^c^
Rutin	1.05 ± 0.03 ^e^	0.07 ± 0.01 ^c^	0.09 ± 0.01 ^d^	0.02 ± 0.00 ^a^	0.07 ± 0.00 ^c^	0.05 ± 0.00 ^b^	0.08 ± 0.01 ^cd^
Apigenin-7-*O*-glucoside	ND	ND	ND	ND	ND	ND	ND
Quercetin	ND	ND	ND	ND	ND	ND	ND
Luteolin	ND	ND	ND	ND	ND	ND	ND
Naringin	2.20 ± 0.02 ^f^	1.05 ± 0.02 ^c^	0.91 ± 0.02 ^a^	1.13 ± 0.02 ^d^	1.12 ± 0.06 ^d^	0.97 ± 0.03 ^b^	1.34 ± 0.06 ^e^
Kaempferol	ND	ND	ND	ND	ND	ND	ND
Apigenin	ND	ND	ND	ND	ND	ND	ND
Isorhamnetin-3-*O*-rutinoside	0.12 ± 0.00 ^d^	0.03 ± 0.00 ^b^	0.04 ± 0.00 ^c^	0.04 ± 0.00 ^c^	0.02 ± 0.00 ^a^	0.02 ± 0.00 ^a^	0.03 ± 0.00 ^b^
Taxifolin	0.28 ± 0.00 ^a^	0.84 ± 0.02 ^c^	1.11 ± 0.05 ^f^	0.98 ± 0.03 ^e^	0.90 ± 0.03 ^d^	0.64 ± 0.02 ^b^	1.00 ± 0.01 ^g^
Isorhamnetin-3-*O*-glucoside	0.20 ± 0.00 ^a^	0.46 ± 0.01 ^c^	0.59 ± 0.02 ^d^	0.56 ± 0.03 ^d^	0.57 ± 0.02 ^d^	0.38 ± 0.01 ^b^	ND
Daidzein	0.03 ± 0.00 ^a^	ND	ND	ND	ND	ND	ND
Eriodictyol	ND	ND	ND	ND	ND	ND	ND
Chrysoeriol	ND	0.03	ND	ND	ND	0.02	ND
Chrysin	ND	ND	ND	ND	ND	ND	ND
Acacetin	ND	ND	ND	ND	ND	ND	ND
Genkwanin	ND	ND	ND	ND	ND	ND	ND
Galangin	ND	ND	ND	ND	ND	ND	ND
Kaempferide	ND	ND	ND	ND	ND	ND	ND
Total	71.04	33.31	33.56	27.88	27.95	22.19	28.15
**Compound**	**Content (mg/L)**						
	**MAE**	**MAE-1**	**MAE-2**	**MAE-3**	**MAE-4**	**MAE-5**	**MAE-6**
Protocatechuic acid	ND *	ND *	ND	ND	ND	ND	ND
*p*-Hydroxybenzoic acid	1.40 ± 0.03 ^a^	2.44 ± 0.02 ^de^	2.35 ± 0.02 ^c^	2.28 ± 0.02 ^b^	2.52 ± 0.06 ^e^	2.41 ± 0.03 ^d^	2.45 ± 0.03 ^de^
Caffeic acid	53.70 ± 0.53 ^b^	41.96 ± 0.12 ^a^	ND	ND	ND	ND	ND
Vanillic acid	1.40 ± 0.01 ^a^	1.72 ± 0.01 ^cd^	1.77 ± 0.03 ^d^	1.64 ± 0.01 ^b^	1.69 ± 0.02 ^c^	1.72 ± 0.02 ^cd^	1.69 ± 0.02 ^c^
Aesculin	3.55 ± 0.03 ^f^	2.13 ± 0.03 ^b^	2.05 ± 0.03 ^a^	2.16 ± 0.02 ^bc^	2.42 ± 0.03 ^e^	2.22 ± 0.03 ^c^	2.26 ± 0.03 ^d^
5-*O*-Caffeoylquinic acid	71.00 ± 0.46 ^g^	22.44 ± 0.15 ^f^	13.68 ± 0.01 ^d^	13.31 ± 0.11 ^c^	14.18 ± 0.11 ^e^	10.27 ± 0.12 ^b^	8.96 ± 0.09 ^a^
*p*-Coumaric acid	10.05 ± 0.11 ^e^	7.24 ± 0.02 ^d^	6.38 ± 0.06 ^a^	6.53 ± 0.06 ^b^	7.29 ± 0.09 ^d^	6.79 ± 0.06 ^c^	6.52 ± 0.06 ^b^
Ferulic acid	6.60 ± 0.09 ^g^	6.15 ± 0.06 ^f^	4.95 ± 0.04 ^d^	4.82 ± 0.06 ^c^	5.50 ± 0.05 ^e^	4.69 ± 0.04 ^b^	4.45 ± 0.04 ^a^
*p*-Hydroxyphenylacetic acid	0.95 ± 0.03 ^a^	1.19 ± 0.04 ^c^	1.08 ± 0.02 ^bc^	1.04 ± 0.03 ^b^	1.14 ± 0.05 ^c^	1.04 ± 0.03 ^b^	1.06 ± 0.02 ^b^
Quercetin-3-*O*-galactoside	0.13 ± 0.01 ^b^	0.02 ± 0.00 ^b^	0.01 ± 0.00 ^a^	ND	0.01 ± 0.00 ^a^	ND	ND
Rutin	2.60 ± 0.08 ^e^	0.30 ± 0.01 ^d^	0.15 ± 0.01 ^c^	0.14 ± 0.02 ^c^	0.15 ± 0.01 ^c^	0.07 ± 0.01 ^b^	0.06 ± 0.00 ^a^
Apigenin-7-*O*-glucoside	0.03 ± 0.00 ^b^	0.02 ± 0.00 ^a^	0.02 ± 0.00 ^a^	0.02 ± 0.00 ^a^	0.03 ± 0.00 ^b^	0.02 ± 0.00 ^a^	0.03 ± 0.00 ^b^
Quercetin	ND	ND	ND	ND	ND	ND	ND
Luteolin	ND	ND	ND	ND	ND	ND	ND
Naringin	3.66 ± 0.06 ^f^	2.33 ± 0.04 ^d^	2.03 ± 0.03 ^a^	2.12 ± 0.03 ^b^	2.19 ± 0.03 ^c^	2.59 ± 0.03 ^e^	2.21 ± 0.03 ^c^
Kaempferol	ND	ND	ND	ND	ND	ND	ND
Apigenin	ND	ND	ND	ND	ND	ND	ND
Isorhamnetin-3-*O*-rutinoside	0.16 ± 0.00 ^c^	0.08 ± 0.00 ^b^	ND	ND	0.04 ± 0.00 ^a^	ND	ND
Taxifolin	0.62 ± 0.00 ^a^	1.88 ± 0.06 ^c^	1.90 ± 0.06 ^d^	1.96 ± 0.05 ^d^	2.18 ± 0.09 ^e^	1.72 ± 0.06 ^b^	1.76 ± 0.05 ^b^
Isorhamnetin-3-*O*-glucoside	0.10 ± 0.00 ^a^	ND	ND	ND	ND	ND	ND
Daidzein	0.08 ± 0.00 ^b^	0.03 ± 0.00 ^a^	ND	ND	ND	ND	ND
Eriodictyol	ND	ND	ND	ND	ND	ND	ND
Chrysoeriol	ND	ND	0.05 ± 0.00 ^a^	ND	ND	ND	ND
Chrysin	ND	ND	ND	ND	ND	ND	ND
Acacetin	ND	ND	ND	ND	ND	ND	ND
Genkwanin	ND	ND	ND	ND	ND	ND	ND
Galangin	ND	ND	ND	ND	ND	ND	ND
Kaempferide	ND	ND	ND	ND	ND	ND	ND
Total	156.03	89.94	36.41	36.02	39.30	33.55	31.42

Values are means of three repeated experiments ± standard deviation. Values in the same row with the different superscript lowercase letters are statistically different (*p* < 0.05). * ND-not detected.

**Table 5:** Antioxidant activity of extract obtained from exhausted leaves.

Sample	Antioxidant Activity		
	IC_50_ (μg/mL)	EC_50_ (μg/mL)	HPMC (%/mL)
	UAE		
UAE	22.25 ± 0.11 ^h^	113.10 ± 0.22 ^l^	53.2 ± 3.3 ^a^
UAE-1	24.64 ± 0.12 ^k^	69.54 ± 0.19 ^h^	92.9 ± 2.1 ^c^
UAE-2	23.71 ± 0.10 ^j^	71.33 ± 0.20 ^j^	118.6 ± 7.8 ^d^
UAE-3	19.71 ± 0.09 ^f^	71.97 ± 0.22 ^k^	117.5 ± 6.9 ^d^
UAE-4	23.10 ± 0.15 ^i^	71.35 ± 0.16 ^j^	80.9 ± 6.4 ^bc^
UAE-5	21.69 ± 0.12 ^g^	66.14 ± 0.19 ^g^	63.9 ± 1.0 ^ab^
UAE-6	25.62 ± 0.12 ^l^	70.44 ± 0.16 ^i^	77.1 ± 4.0 ^bc^
	MAE		
MAE	18.92 ± 0.13 ^e^	69.36 ± 0.11 ^h^	148.0 ± 8.2 ^efg^
MAE-1	13.08 ± 0.11 ^ab^	60.54 ± 0.21 ^e^	164.0 ± 5.3 ^g^
MAE-2	12.86 ± 0.09 ^a^	59.27 ± 0.16 ^c^	143.2 ± 6.6 ^ef^
MAE-3	17.41 ± 0.10 ^c^	59.90 ± 0.25 ^d^	151.9 ± 4.5 ^efg^
MAE-4	18.09 ± 0.11 ^d^	58.62 ± 0.23 ^b^	163.4 ± 9.1 ^g^
MAE-5	13.29 ± 0.15 ^b^	62.97 ± 0.29 ^f^	159.6 ± 9.2 ^fg^
MAE-6	17.61 ± 0.15 ^c^	55.70 ± 0.33 ^a^	141.8 ± 5.7 ^e^

Values are means of three repeated experiments ± standard deviation. Values in the same column with the different superscript lowercase letters are statistically different (*p* < 0.05).

**Table 6:** Cytotoxic activity of extract obtained from exhausted leaves.

Sample	Cell Line/IC_50_ (μg/mL)		
	Hep2c	RD	L2OB
	UAE		
UAE	36.36 ± 0.12 ^i^	29.67 ± 0.34 ^g^	39.16 ± 0.32 ^i^
UAE-1	35.45 ± 0.39 ^i^	32.49 ± 0.98 ^h^	36.23 ± 0.76 ^h^
UAE-2	31.29 ± 0.83 ^h^	29.24 ± 0.38 ^g^	33.19 ± 0.43 ^g^
UAE-3	29.46 ± 0.54 ^g^	26.76 ± 0.83 ^f^	28.76 ± 0.64 ^e^
UAE-4	23.39 ± 0.72 ^e^	20.84 ± 0.16 ^d^	26.48 ± 0.13 ^d^
UAE-5	13.35 ± 0.15 ^b^	11.39 ± 0.30 ^b^	14.60 ± 0.08 ^c^
UAE-6	9.23 ± 0.67 ^a^	8.72 ± 0.23 ^a^	13.13 ± 0.32 ^b^
	MAE		
MAE	38.47 ± 0.43 ^j^	31.32 ± 0.54 ^h^	49.28 ± 0.45 ^j^
MAE-1	25.28 ± 0.20 ^f^	23.73 ± 0.67 ^e^	36.43 ± 0.18 ^h^
MAE-2	23.29 ± 0.82 ^e^	20.89 ± 0.29 ^d^	32.39 ± 0.38 ^fg^
MAE-3	20.43 ± 0.14 ^d^	18.18 ± 0.56 ^c^	31.45 ± 0.24 ^f^
MAE-4	18.34 ± 0.30 ^c^	17.35 ± 0.91 ^c^	25.83 ± 0.92 ^d^
MAE-5	14.49 ± 0.29 ^b^	11.08 ± 0.18 ^b^	15.65 ± 0.15 ^c^
MAE-6	10.17 ± 0.89 ^a^	9.46 ± 0.37 ^c^	11.30 ± 0.27 ^a^

Values are means of three repeated experiments ± standard deviation. Values in the same column with the different superscript lowercase letters are statistically different (*p* < 0.05).

## Data Availability

Not applicable.

## Supplementary Materials

The following supporting information can be downloaded at: https://www.mdpi.com/article/10.3390/foods12040809/s1, Figure S1: UV chromatograms of all samples recorded at 280 nm, Table S1: Correlation analysis of the antioxidant and cytotoxic activity of extract obtained from exhausted leaves, Table S2: Antimicrobial activity of UAE extracts, Table S3: Antimicrobial activity of MAE extracts
