# Supplementary material for: Recovery of Biologically Active Compounds from Stinging Nettle Leaves Part II: Processing of Exhausted Plant Material after Supercritical Fluid Extraction
Source: Foods. 2023 Feb 14;12(4):809. doi: 10.3390/foods12040809 (PMC9957156; doi:10.3390/foods12040809)
Supplement: Supplementary file 1 [file foods-12-00809-s001.zip › foods-2170406-supplementary.pdf]

Recovery of biologically active compounds from stinging nettle leaves part II: processing of exhausted plant material after supercritical fluid extraction

Saša Đurović[a,b,*], Lato Pezo[a], Uroš Gašić[c], Stanislava Gorjanović[a], Ferenc Pastor[d], Julia G. Bazarnova[b], Yulia A. Smyatskaya[b], Zoran Zeković[e]

[a]Institute of General and Physical Chemistry, Studentski trg 12/V, 11158 Belgrade, Serbia

[b]Peter the Great Saint-Petersburg Polytechnic University, Graduate School of Biotechnology and food industries, Polytechnicheskaya street, 29, 195251 Saint-Petersburg, Russia

[c]Institute for Biological Research "Siniša Stanković" – National Institute of Republic of Serbia, University of Belgrade, Bulevar despota Stefana 142, 11060 Belgrade, Serbia

[d]University of Belgrade, Faculty of Chemistry, Studentski trg 12, 11000 Belgrade, Serbia

[e]University of Novi Sad, Faculty of Technology, Bulevar Cara Lazara 1, 21000 Novi Sad, Serbia

*Correspondence:

*Laboratory of Chromatography, Institute of General and Physical Chemistry, Studentski trg 12/V, 11158 Belgrade, Republic of Serbia, Tel: +381659577200

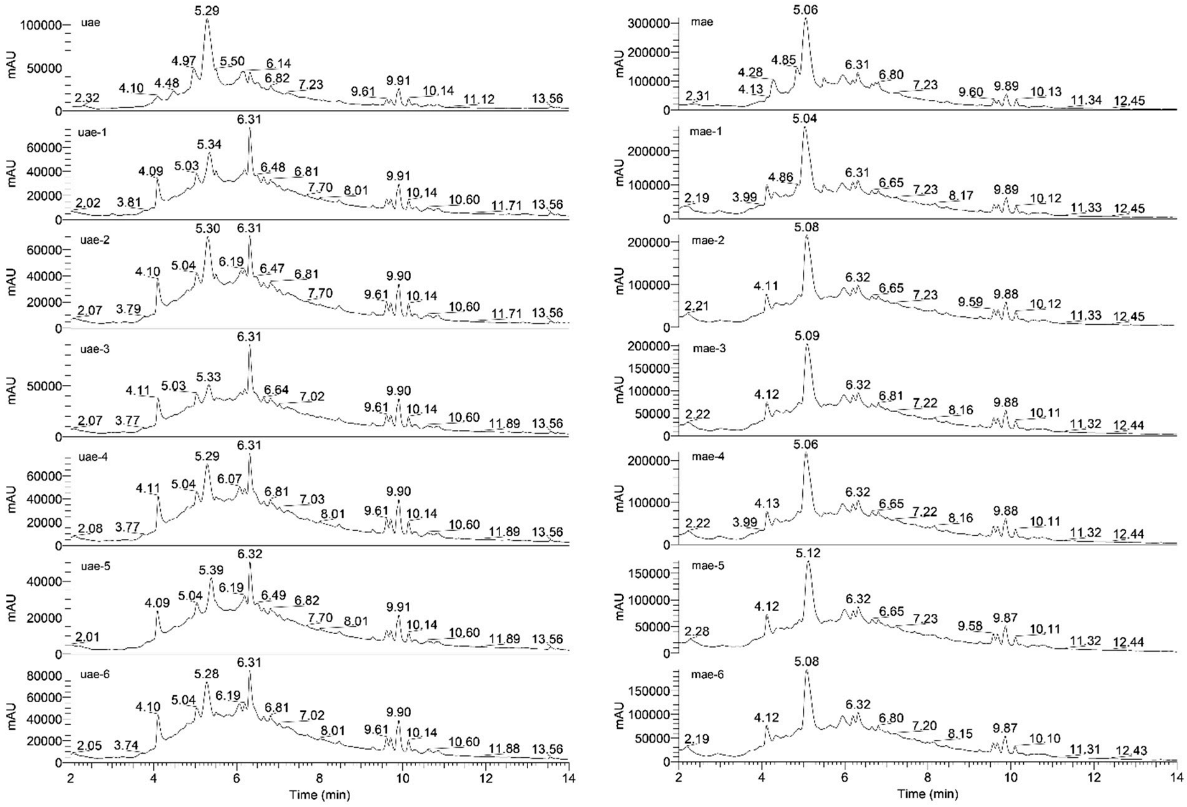

Figure S1. UV chromatograms of all samples recorded at 280 nm

Table S1. Correlation analysis of the antioxidant and cytotoxic activity of extract obtained from exhausted leaves

	EC50	HPMC	Hep2c	RD	L2OB	TPC	TFC
IC50	0.460**	-0.787+	0.178	0.199	-0.036	-0.875++	-0.856++
EC50		-0.678+	0.521**	0.453	0.349	-0.630*	-0.655*
HPMC			-0.101	-0.078	0.124	0.783+	0.796+
Hep2c				0.987++	0.923++	-0.377	-0.397
RD					0.909++	-0.408	-0.419
L2OB						-0.093	-0.105
TPC							0.993++

Correlation was statistically significant at: ++p<0.001 level; +p<0.01 level; *p<0.05 level; **p<0.10 level.

Table S2. Antimicrobial activity of UAE extracts

Microbial strains	Sample / MIC (µg/mL)						
	UAE	UAE-1	UAE-2	UAE-3	UAE-4	UAE-5	UAE-6
Staphylococcus aureus (*ATCC 25923*)	125.00	125.00	62.50	125.00	31.25	31.25	15.82
Klebsiella pneumoniae (*ATCC 13883*)	250.00	125.00	250.00	31.25	62.50	62.50	15.82
Escherichia coli (*ATCC 25922*)	62.50	250.00	250.00	31.25	125.00	15.82	31.25
Proteus vulgaris (*ATCC 13315*)	62.50	125.00	125.00	31.25	15.82	15.82	31.25
Proteus mirabilis (*ATCC 14153*)	500.00	62.50	62.50	31.25	125.00	62.50	7.81
Bacillus subtilis (*ATCC 6633*)	250.00	125.00	500.00	125.00	62.50	125.00	31.25
Candida albicans (*ATCC 10231*)	500.00	250.00	31.25	125.00	125.00	15.82	7.81
Aspergillus niger (*ATCC 16404*)	500.00	250.00	125.00	500.00	62.50	62.50	7.81

Table S3. Antimicrobial activity of MAE extracts

Microbial strains	Sample / MIC (µg/mL)						
	MAE	MAE-1	MAE-2	MAE-3	MAE-4	MAE-5	MAE-6
Staphylococcus aureus (*ATCC 25923*)	62.50	62.50	31.25	31.25	15.82	7.81	15.82
Klebsiella pneumoniae (*ATCC 13883*)	125.00	125.00	62.50	31.25	62.50	31.25	15.82
Escherichia coli (*ATCC 25922*)	125.00	62.50	125.00	62.50	62.50	15.82	31.25
Proteus vulgaris (*ATCC 13315*)	62.50	125.00	125.00	31,25	15.82	15.82	31.25
Proteus mirabilis (*ATCC 14153*)	31.25	62.50	125.00	31.25	15.82	62.50	7.81
Bacillus subtilis (*ATCC 6633*)	62.50	125.00	62.50	31.25	62.50	31.25	15.82
Candida albicans (*ATCC 10231*)	250.00	125.00	125.00	62.50	31.25	15.82	31.25
Aspergillus niger (*ATCC 16404*)	125.00	31.25	62.50	125.00	62.50	31.25	7.81
